# The wash-in effect and its significance for mass casualty decontamination

**DOI:** 10.1080/10937404.2022.2042443

**Published:** 2022-02-27

**Authors:** Thomas James, Lydia Izon-Cooper, Samuel Collins, Haydn Cole, Tim Marczylo

**Affiliations:** a Radiation, Chemical and Environmental Hazards, UK Health Security Agency, Chilton, UK; b Global Operations, UK Health Security Agency, London, UK

**Keywords:** Emergency response, chemical, wash-in effect, skin penetration, casualty decontamination

## Abstract

Decontamination of skin by washing may increase dermal absorption, a phenomenon known as the wash-in effect. The wash-in effect is frequently discussed in studies investigating casualty decontamination where potentially life-saving interventions may enhance the dermal penetration of toxic chemicals, leading to an increase in incidence of morbidity and rates of mortality. However, the wash-in effect is seldom investigated within the context of mass casualty decontamination and real-life consequences are therefore poorly understood. This paper reviews the existing literature on the wash-in effect to highlight the proposed mechanisms for enhanced absorption and evaluate the wash-in effect within the context of mass casualty chemical decontamination.

## Introduction

Chemical incidents involving the general public require casualty decontamination methods including dry or wet, improvised, interim, or mass decontamination, during both the Initial and Specialist Operational Response (IOR and SOR respectively) of the UK CBRN Response Framework (Home Office 2015). While dry decontamination is recommended during IOR, wet decontamination is used when a more structured, specialist response is available, including a ladder pipe system (two fire engines and hoses creating a corridor with fixed showering positions) as part of interim decontamination, and dedicated mass decontamination units for SOR. Wet decontamination can also be implemented during the IOR in situations involving corrosive compounds. A concern regarding the use of water for decontamination is a potential increase in the dermal penetration of chemicals. Dermal interactions with chemicals involving water are well documented both *in vivo* and *in vitro*, with studies identifying enhanced penetration of chemicals as a possible result of skin hydration as early as 1977 (Wester, Noonan, and Maibach 1977), and a “washing-in effect” being described in 1985 (Wester and Maibach 1985). Following this, the term ‘wash-in effect’ was introduced by Moody and Nadeau (1993) in a series of *in vitro* studies investigating the penetration of N,N-diethyl-*m*-toluamide (DEET) through skin. Moody and Maibach (2006) reviewed evidence for the wash-in effect focussing on a series of their own studies in which both *in vivo* and *in vitro* penetration of chemicals were determined, and wash-in effects identified under some, but not all, *in vitro* conditions. Moody and Maibach (2006) concluded that, under certain circumstances, such as use of surfactants or increased friction during decontamination, the act of washing contaminated skin leads to enhanced dermal penetration. This is a concern for first responders, as an increase in the dermal penetration of hazardous chemicals may elevate the risk or severity of harm. While previous literature reviews identified both *in vivo* (Burli et al. 2021) and *in vitro* (Chiang et al. 2021a) decontamination studies that displayed wash-in effects, no apparent reviews have specifically investigated the wash-in effect since 2006. This literature review (1) assesses the current published information available to better understand this phenomenon, (2) describes the proposed mechanisms, and (3) explores whether the wash-in effect has implications for decontamination interventions.

## Materials and methods

Two simultaneous literature searches were conducted to ensure both unidentified instances and author-identified instances of the wash-in effect were detected. The first focused on the enhanced penetration of chemicals through skin as a result of interactions with water, and the second specifically on the wash-in effect.

Search terms were identified through selection of keywords from existing literature. Topics such as skin penetration and skin hydration were combined as the foundation of the main search. The wash-in literature search consisted of a simple searching for the term “wash-in effect.” Final searches are shown in Appendix A.

All forms of publications, including gray literature, letters, and peer-reviewed articles were included in the search, except from previous literature reviews on the topic, which were discussed but not included in the results due to providing no primary data. Databases searched were Medline through Ovid Technologies Inc., Scopus, and EMBASE and PubMed in a combined search through the Healthcare Databases Advanced Search (HDAS). All searches were temporally unrestricted and conducted in June 2020 with an automatic monthly update to identify relevant, newly published papers. The final date of automatic update was 2^nd^ December 2021.

All fields of the publication were included in the search (e.g. title, abstract, and full text). During screening, if key words such as “wash-in effect” or “hydration” or “skin penetration” or similar were not initially obvious from the title or the abstract, the paper was discounted. Following title and abstract screening, papers were assessed for relevance. Papers specifically focussing on nanoparticles, drug delivery, penetration enhancing chemicals and biological agents were discounted. During the full-text appraisal stage, references of each paper were screened to identify any not picked up by the systematic searches. Bias was minimized by conducting the review across databases (with appropriate changes to syntax) and assessing all identified literature in the same manner in line with the updated 2020 PRISMA methodology (Page et al. 2021).

## Results

**Figure 1.**
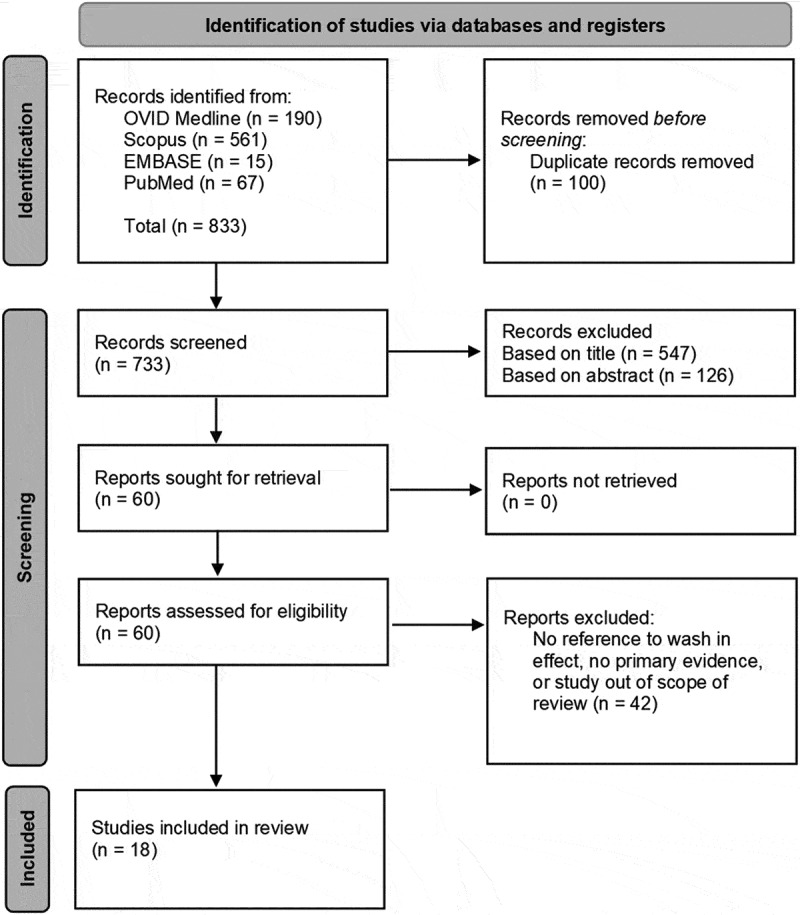
The PRISMA flow diagram showing the stages of screening and exclusion. From 733 unique papers, 60 full studies were assessed with 18 being included in the review.

Figure 1 illustrates the combined results of the literature reviews, and the PRISMA process undertaken during literature screening. Table 1 contains a brief summary of the 18 identified studies, evidence contained within for the wash-in effect, and the proposed mechanisms suggested by the investigators. Most studies reported only data generated *in vitro* using diffusion cell studies of *ex-vivo* skin, one study noted only *in vivo* data, one reported on data from human volunteers using sequential tape stripping to determine penetration, and 5 compared data from both *in vivo* and *in vitro* investigations. In the latter 5, no wash-in effect was identified *in vivo*. For clarity, Table 2 presents each proposed mechanism for the wash-in effect and the studies that mentioned each mechanism.

**Table 1.** Combined results of the literature searches

Reference	Model and summary of study	Evidence of the wash-in effect	Proposed mechanism(s) by authors
Brand et al. 2007	An *ex vivo* (mouse skin) assessment of altered herbicide skin penetration as a result of moisturization and hand washing	Washing skin with 5% SLS prior to 2,4-D exposure increases its percutaneous penetration from 43.4 ± 3.7% to 62.9 ± 9.5% (p < .01).	SC hydration effects Surfactant effects
Dalton, Graham, and Jenner 2018	An *in vitro* (porcine skin) diffusion cell assessment of VX penetration through skin in the presence of water	Penetration rates of 10 µl neat VX measured through wetted skin (86 ± 52 µg cm^−2^ h^−1^) between 1 and 3 h were significantly (p < .05) higher than through dry skin (19 ± 23 µg cm^−2^ h^−1^).	Physical effects Physicochemical effects
Forsberg et al. 2020	An *in vitro* (human skin) assessment of soap water and water decontamination of TICs and CWA simulants	An unquantified temporary increase in the penetration rate of 10 µl methyl salicylate through skin occurred directly after washing with either water or soapy water. An increase for 2-butoxyethanol was only observed after soapy water decontamination.	SC hydration effects Surfactant effects pH effects
Lademann et al. 2011	A human study into dry vs wet decontamination and their impact on follicular penetration	Using tape strips to determine depth of penetration, octylmethoxycinnamate was detected more deeply into the skin by washing (~7 cell layers) than by decontamination with dry absorbent materials (~ 3 cell layers).	Physical effects
Loke et al. 1999	An *in vitro* (human skin) study into stratum corneum hydration induced chemical penetration by various wet decontamination interventions	Wet decontamination conducted at 05h and 1h enhanced the penetration rate of diethyl malonate through skin in the immediate 2 h following decontamination when compared to no-decontamination control. A mean penetration rate enhancement of 105.55 µg h^−1^ was displayed when decontaminating skin with water at 1 h over the control.	SC hydration effects Physical effects
Matar et al. 2016	An *in vitro* (porcine skin) evaluation of a range of decontamination products on the removal of methyl salicylate	Five water-containing products enhanced the rate and amount of penetration of methyl salicylate through skin. Over a no-decontamination control penetration of 2.5 ± 1.2%, diphoterine increased receptor fluid percentage to 6.2 ± 3.4% while baby wipes increased percentage up to 10.1 ± 5.5% over a 24 h period.	SC Hydration effects Surfactant effects
Misik et al. 2012	An *in vitro* (porcine skin) assessment of decontamination procedures on the penetration of paraoxon.	The penetration of paraoxon through wet skin was significantly (p = .04) higher than through dry skin, with no significant difference between dry, cold and warm skin. After 24h on dry skin, skin permeation of paraoxon was four times higher after a 3 min shower, and 60% higher after a 30s shower than controls.	Surfactant effects Physical effects *In vitro* artifact
Moody and Nadeau 1993	An *in vivo/in vitro* (mouse, rat, Guinea pig, pig, human, and tissue culture skin) comparison study into the dermal penetration of DEET.	Unquantified temporal increases in penetration rates were seen for rat, Guinea pig, pig and Testskin, but not in human skin. The temporal increase occurs following a soap water wash at 24h. No in vivo wash-in effect was identified.	Surfactant effects
Moody and Nadeau 1994	An *in vivo/in vitro* (rat, Guinea pig, pig, human, and tissue culture skin) comparison study into the dermal penetration of diazinon.	Unquantified temporal increases in penetration rates of diazinon were seen for rat, Guinea pig, pig, human and Testskin skin following a soap water wash at 24h, however penetration over time is only provided for human and Testskin data. No in vivo wash-in effect identified.	Surfactant effects
Moody and Nadeau 1997	An *in vivo/in vitro* (rat, Guinea pig, and human skin) comparison study into the dermal penetration of 2,4-D	A prominent peak in the amount of 2,4-D dermal penetration was seen for each skin at 26h post exposure, 2h after a 24h soap water wash. Following the temporal peak, increased rates of penetration did not continue to decline appreciably, remaining above baseline levels. No in vivo wash-in effect identified.	SC hydration effects Surfactant effects pH effects *In vitro* artifact
Moody, Nadeau, and Chu 1994	An *in vivo/in vitro* (rat, Guinea pig, pig, human, and tissue-cultured skin) comparison study into the dermal penetration of DDT.	Unquantified temporal increases in penetration rate of DDT were seen for rat and human skin following a soap water wash at 24h. No in vivo wash-in effect identified.	Surfactant effects Physical effects
Moody, Nadeau, and Chu 1995	An *in vivo/in vitro* (rat, Guinea pig, pig, and human skin) comparison study into the dermal penetration of DEET.	Up to a 32-fold increase in DEET penetration was seen *in vitro* for each skin at 26h, 2h post-water wash. No in vivo wash-in effect identified.	Surfactant effects
Thors et al. 2020	An *in vitro* (human skin) comparison between RSDL and wet decontamination in the removal of VX.	A significant (p < .05) but unquantified increase in maximum penetration rate of VX through skin was seen 1.5h following soapy water decontamination when compared with no-decontamination control. The cumulative penetrated amount of VX was also significantly (p < .05) increased when using soapy water decontamination (122.4 ± 22.3 µg cm^−2^) compared to the control without decontamination (56.0 ± 6.7 µg cm^−2^).	SC hydration effects Surfactant effect Physicochemical effects
Thors, Wigenstam 2021	An *in vitro* (human skin) comparison between RSDL and wet decontamination in the degradation of VX on and within skin.	Soapy water decontamination caused a significant (p < .05) increase in the amount of VX penetration through skin (0.87 ± 0.18 µg cm^−2^) compared to no-decontamination control (0.26 ± 0.07 µg cm^−2^), but significantly (p < .05) decreased agent amounts found within the stratum corneum.	Surfactant effects
Thors, Wästerby 2021	An *in vitro* (human skin) comparison between RSDL and wet decontamination on the removal and degradation of VX on human skin under varying cold environmental conditions.	Following application of 10 µl VX to skin and soapy water decontamination at room temperature, a significant (P < .001) increase in cumulative amounts penetrated was seen compared to no-decontamination controls. When this was repeated at −5°C and −15°C ambient air temperatures however, cumulative penetration did not significant differ from no decontamination controls.	Physical effects
Wester, Noonan, and Maibach 1977	An *in vivo* (Rhesus monkey) determination of hydrocortisone penetration as a single or multiple dose application, with and without washing.	A significantly higher (p < .05) percentage dose of hydrocortisone was excreted between 0–24h in soapy water wash condition (0.17%) when compared against the no wash control (0.06%).	SC hydration effects
Yousef et al. 2017	An *in vitro* (human skin) study into hydration enhanced dermal penetration of salicylates.	Following application of both neat and dilute solutions of ethyl, methyl and glycol salicylate to hydrated or dry skin, enhancements in rates of epidermal penetration between 1.95–10 fold (neat) and 1.98–2.32 fold (solutions) were observed.	SC hydration effects *In vitro* artifact
Zhu et al. 2015	An *in vitro* (human skin) study to identify the wash-in effect by measuring the penetration of four chemicals at varying levels of skin hydration.	A significant, cumulative enhanced penetration of paraoxon was reported through highly hydrated skin, (p = .001) after a soap water wash (3.1 ± 1.2%) compared to the non-wash control (1.2 ± 0.2%) (n = 4). The cumulative amount of benzoic acid that penetrated was lower when washed, but the peak dose 30 min post wet decontamination is around 3-fold higher than the non-wash control. No wash-in effect was seen for clonidine or hydroquinone.	SC hydration effects Physicochemical effects

**Table 2.** Each identified study listed by the proposed wash-in effect mechanism

Proposed wash-in effect mechanism by author	Study
SC hydration effects	Brand et al. 2007 Forsberg et al. 2020 Loke et al. 1999 Matar et al. 2016 Moody and Nadeau 1997 Thors et al. 2020 Wester, Noonan, and Maibach 1977 Yousef et al. 2017 Zhu et al. 2015
Physical effects	Dalton, Graham, and Jenner 2018 Lademann et al. 2011 Loke et al. 1999 Misik et al. 2012 Moody, Nadeau, and Chu 1994 Thors et al. 2021a
Surfactant effects	Brand et al. 2007 Forsberg et al. 2020 Matar et al. 2016 Misik et al. 2012 Moody and Nadeau 1993 Moody and Nadeau 1994 Moody and Nadeau 1997 Moody, Nadeau, and Chu 1994 Moody, Nadeau, and Chu 1995 Thors et al. 2020 Thors et al. 2020
Physicochemical effects	Dalton, Graham, and Jenner 2018 Thors et al. 2020 Zhu et al. 2015
pH effects	Forsberg et al. 2020 Moody and Nadeau 1997
In vitro artifacts	Misik et al. 2012 Moody and Nadeau 1997 Yousef et al. 2017

## Discussion

Moody and Maibach (2006) proposed 5 distinct possible mechanisms driving the wash-in effect: (1) hydration of the skin producing disruption to the structure of skin through the swelling of corneocytes, (2) surfactants initiating delipidation and membrane fluidization able to free reservoirs of pooled compound, (3) physical interaction with the skin including friction from rubbing, (4) acid/base disruption to the skin including the acid-mantle, and (5) artifact-effects that proposed that the wash-in effect may be as a result of the predominantly *in vitro*, diffusion cell methodology being utilized for the published studies. Previously other investigators came to similar conclusions (Loke et al. 1999; Wester, Noonan, and Maibach 1977) and subsequent studies have broadly attributed the wash-in effect to these 5 mechanisms, further evidenced by a recent review into *in vitro* wet decontamination studies (Chiang et al. 2021b) that identified four instances of the wash-in effect. The studies identified in *this* review were conducted for a range of purposes, from investigating handwashing and moisturizing (Brand et al. 2007) to investigating the decontamination of chemical warfare simulants (Forsberg et al. 2020; Matar et al. 2016) and highly toxic chemical agents (Dalton, Graham, and Jenner 2018; Thors et al. 2020).

**Figure 2.**
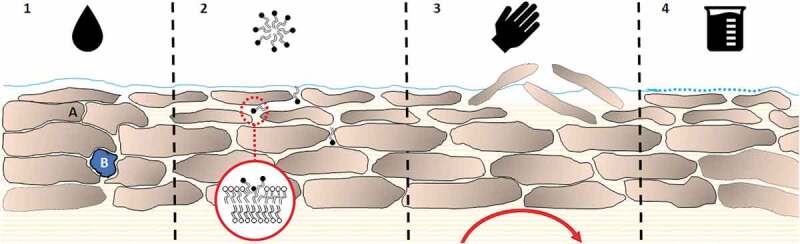
A model skin diagram outlining four of the proposed mechanisms for the wash-in effect (physicochemical effect and *in vitro* artifact not shown as no harmonized single mechanism). 1) The effect of hydration on skin with (A) showing the swelling corneocytes causing cavitation between cells which leads to (B) pooling of water within the SC. SC hydration is also intrinsically linked with thermodynamic effects including altered diffusion of chemicals within the SC. 2) Surfactant micelles penetrate through the lipid bilayer, eventually leading to delipidation and membrane fluidization. 3) Physical effects including friction can remove external layers of the SC, compromising the barrier function and increasing penetration. Blood flow can further increase systemic absorption through rubefacient action, increasing clearance of compound within the skin. 4) Acids or bases can alter the acid mantle on the skin, compromising the skins barrier.

Through this review of the proposed mechanisms for the wash-in effect, six categories were broadly separated (Figure 2). (1) **Hydration effects**, including simple hydration of the stratum corneum (SC), (2) **physical effects** such as desquamation, friction, temperature, (3) **surfactant effects** including release of the dermal depot by interaction with soaps, (4) **pH effects** induced by changes in skin pH, (5) **physicochemical effects** governed by specific physicochemical properties and finally (6) any ***in vitro* artifacts**.

One influencing factor not included here, but important to the understanding and interpretation of skin penetration and the wash-in effect, is the influence of thermodynamic effects. This includes the concentration-based thermodynamic equilibrium governing the permeation of compounds (especially lipophilic compounds) into the SC, the thermodynamic gradient governing diffusion between the vehicle and into/through the SC and ultimately increased absorption as a result of addition of a water vehicle to the skin.

Very few studies identified in this review make explicit reference to the influence of thermodynamic effects. Dalton, Graham, and Jenner (2018) postulated that due to the non-linear relationship between concentration of penetrant and penetration through skin, the presence of water is changing the permeability coefficient of the SC. While this study did not investigate which parameter within the permeability coefficient was being altered, Dalton, Graham, and Jenner (2018) suggested hydration may exert an impact on both diffusion coefficient and diffusional path length. Yousef et al. (2017) also identified that compounds with varying physicochemical properties display differing magnitudes of enhancement in skin permeation when applied in a saturated solution, and neat hygroscopic compounds might further enhance skin penetration through water uptake into the SC and increasing diffusivity. Beyond these studies, no explicit reference is made to thermodynamic effects by the manuscripts captured in this review. An in depth understanding of the thermodynamic effects affecting hydration of skin and enhanced chemical penetration during mass decontamination therefore represents a knowledge gap as thermodynamic effects are influencing the wash-in effect in the majority of studies identified in this review. To better understand the relationship between thermodynamic effects and the wash-in effect, additional investigations will need to be conducted, determining the effect of hydration on each thermodynamic parameter.

### Hydration effects

The degree of dermal absorption heavily relies on the barrier function of the SC (Anderson and Meade 2014), and when this is compromised by factors such as hydration or structural damage the barrier function might decrease. While not always the case (Behl *et al*. 1982), under certain conditions the SC can absorb water, effectively changing its physical structure and impeding its ability as a barrier to both in- and egress of certain compounds (Blattner et al. 2014; Zhang et al. 2006). This complex process occurs when water enters corneocytes, swelling them and forming liquid “pools” and inter-corneocyte ruptures within the skin structure (Tan et al. 2010) that opens up the compact SC by increasing the amount of unbound water in the SC. These interactions disorder the structure of the lipids and proteins within the SC (Grice et al. 2017) and subsequently reduces the barrier effectivity. Wester, Noonan, and Maibach (1977) conducted a study on Rhesus monkeys (n = 3 to 5) in which the penetration of three doses (13.3 µg/cm^2^) of hydrocortisone applied to the ventral forearm was measured both with and without soap water washes between applications. When soap water washes were conducted, a significantly larger amount of hydrocortisone was excreted in urine in the first 24 h (0.17%, no measures of variability provided) than the non-wash experiment (0.06%). Wester, Noonan, and Maibach (1977) concluded that the soap-wash may have hydrated the skin, increasing skin absorption. This study does not describe the experimental detail however, so the methodology cannot be assessed, and while the results are significant, *in vivo* replicates within studies might be highly variable. Further, as soap water was used the relative contributions of hydration and surfactant (see below) mechanisms cannot be determined.

Brand et al. (2007) showed that artificially increasing moisture in the SC using moisturizing lotions might also enhance the penetration of chemicals. While this facet of their study was not directly linked to an investigation of the wash-in effect, data show the effect that skin hydration might exert on dermal penetration. The study demonstrated that when a commercially available body lotion was applied to excised hairless mouse skin, hydration remained elevated in the SC for 4 hr before returning to baseline levels, while deeper in the skin, hydration remained significantly raised for at least 6 hr. This rise in hydration led to a significant increase in penetration of the herbicide 2,4 dichlorophenoxyacetic acid (2,4-D) solution (66.3 ± 8.8%) over control (39.1 ± 3%). The chemical composition of the lotion was not reported, therefore the exact mechanism producing the enhanced penetration is unclear (ions in the lotions may enhance moisture measurements), however elevated SC water content correlated with enhanced penetration.

More recently, an investigation into the penetration rates of benzoic acid, paraoxon, hydroquinone, and clonidine when applied to SC with varying hydration states (0–295%) and subjected to surface washing with water (3 × 1 ml water for 10 s) (Zhu et al. 2015) showed SC penetration of both benzoic acid and paraoxon was found to rise with increasing SC hydration levels, a phenomenon that these investigators might also be attributed to the increase in capacity of the SC reservoir due to hydration. The same effect was not seen for either hydroquinone or clonidine. These findings were replicated in viable epidermis using cotton balls soaked in either water or 3% soap (3 min wash with no wiping), in which a threefold temporary enhancement to the rate of epidermal penetration was seen for benzoic acid, and a two-fold enhancement was seen for paraoxon, but no significant change was noted for hydroquinone or clonidine. This study highlights both the temporal and chemical-specific nature of the wash-in effect. The former may explain why an earlier *in vitro* study (Nielsen 2010) did not detect a wash-in effect using a dilute formulation of benzoic acid (107 µl of 4 mg/ml) due to the relatively long periods between receptor fluid collections (2 hr). This demonstrates that experimental design and timeliness of sample collection to identify the wash-in effect is a critically important consideration.

The enhancing effect of SC hydration was also investigated by Loke et al. (1999). Part of the study focussed on the effect water had on the removal of a finite dose (as defined by the Organization for Economic Co-operation and Development, OECD 2004) (4 µl) of diethyl malonate from human skin *in vitro* when wet decontamination was conducted at 15 min, 30 min and 1 hr post-exposure. The deionized water decontamination resulted in a mean elevation in rate of penetration of 105.55 µg/hr (SEM 30.77, n = 3) over the non-decontaminated control (mean penetration rate 99.21 ± 16.46 µg/hr, n = 3). Loke et al. (1999) suggested that the enhancement in chemical penetration following wet decontamination might be attributed to the temporary skin hydration leading to a reduction in skin barrier properties initiated during washing, however it is important to add that this study was under-powered and the increase in penetration rate is not significant. Additional studies were conducted in which saline solutions (0.9% w/v and 9% w/v) were used for decontamination. These isotonic and hypertonic solutions showed a mean enhancement in penetration rate of 71.27 µg/hr (SEM 22.85, n = 3) and 23.17 µg/hr (SEM 20.84, n = 3), respectively, both less than that of deionized water. This reduction in mean enhanced penetration rates produced by increasing salinity is not fully explained in the investigation, however the lack of statistical significance means that further, more appropriately powered, studies are required.

The potential role of osmolarity in facilitating the wash-in effect raises the question whether sweat might also influence skin penetration of chemicals. Dalton et a (2018) aimed to investigate this by assessing the penetration of VX through porcine skin in the presence and absence of water or artificial sweat under finite dosing conditions. In the study, the 10 µl droplet of VX penetrated significantly faster between 1 and 3 hr when applied to skin pre-wetted with 200 µl water (maximum rate 86 ± 52 µg/cm^2^^/^hr) than through dry skin (maximum rate 19 ± 23 µg/cm^2^^/^hr). Values given are mean ± standard deviation of up to n = 8 replicates. Interestingly, there was no significant difference between the dry condition and the condition in which the skin had been pre-wetted with 200 µl of artificial sweat (maximum rate 48 ± 34 µg/cm^2^^/^hr). Data suggested that the discrepancy between artificial sweat and water may be due to the presence of metabolites, minerals, and amino acids in the sweat. The physiological saline at 0.9% used in the study by Loke would have equated to 154 mmol/L, approximately threefold higher than that of the average sweat concentration of sodium (approximately 40–60 mmol/L) (Turner and Avolio 2016) which, while not stated, would presumably be present in the eccrine sweat used by Dalton, Graham, and Jenner (2018). Both sweat and saline showed reductions in the penetration rate, indicating that osmolarity may indeed be affecting chemical transfer by altering the hydration of the skin. Hypertonic solutions may induce a net flow of water out of cells, whereas hypotonic solutions (including water) might result in a net increase in hydration of the SC. However, due to the different study conditions, dosing agents, and methodological design, further research would need to be conducted to determine whether the effect of salinity, especially that of sweat, exerts any effect on the wash-in effect during human decontamination. Both studies agree that there may be additional factors influencing the wash-in effect, including enhanced spreading of the dosing agent across the skin leading to an enhancement through increased surface area and penetration.

### Physical effects

Loke et al. (1999) proposed that the wash-in effect can be attributed to the physical spreading of diethylmalonate during washing, effectively increasing the skin surface contact area and consequently increasing the rate of penetration. In addition, Dalton, Graham, and Jenner (2018) noted that within 3 hr of VX application to pre-wetted skin (200 μl water) the 10 μl droplet of VX had spread more rapidly than under the other study conditions. This elevation in spread likely contributed to more rapid uptake of VX through the skin due to higher surface area. Penetration rate through pre-wetted skin was enhanced significantly between 1 and 3 hr, but not between 12 and 24 hr, an observation that might be attributed to the fact that by 12 hr the surface spread under all conditions was comparable.

Spreading of dosing agent was also noted by Misik et al. (2012) using paraoxon. When 10 µl of undiluted paraoxon was applied to cold, warm, and dry skin, the agent remained as a droplet around 0.16 cm^2^ in size for 24 hr. When applied to wet skin however the droplet spread out, increasing surface area (magnitude not stated). The penetration through wet skin was found to be significantly higher than through dry skin, while dry, cold, and warm skin were not significantly different. The incomparable decontamination conditions of 0.5 ml/min water and the fact that decontamination efficacy was based upon cumulative penetration rather than flux however mean that other than the increase in surface area it is difficult to identify a sole main cause for the wash-in effect.

Investigations that suggest mechanical disruption effects are a contributing factor to the wash-in effect include Moody, Nadeau, and Chu (1994) where a more pronounced *in vitro* wash-in effect of DDT was detected in diffusion cells containing rat or human skin that underwent swabbing compared to simple soap water perfusion. Laddemann *et al*. (2011) observed a reduction in surface contamination with octylmethoxycinnamate but deeper SC penetration when washing and manually massaging the skin compared to non-decontamination control.

While most studies occurred at room temperature (approximately 25°C), Thors et al. (2021a) also conducted soapy water decontamination at −5°C and −15°C. While the cumulative penetration of VX significantly increased (66.5 ± 5.5 μg/cm^2^ compared to no-decontamination control of 24.6 ± 6.6 μg/cm^2^) following wet decontamination at room temperature as previously identified (Thors et al. 2021b, 2020), there was no significant alteration at either −5°C (36.0 ± 3.7 μg/cm^2^compared to 28.9 ± 8.9 μg/cm^2^) or −15°C (35.8 ± 9.4 μg/cm^2^ompared to 34.4 ± 5.3 μg/cm^2^). Skin penetration of VX was significantly reduced at lower temperatures when compared to room temperature, although this is based upon cumulative dose over 5 hr, and presence of any temporal phenomenon is not mentioned within the study. Thors et al. (2020); (2021b)) suggested that the lack of wash-in effect at lower temperatures may be due to freezing of water on the surface of the skin (despite the skin being held at 32°C) and that cold soapy water reaching into the skin may have delayed further VX penetration within the skin. While this is an important consideration for incidents that occur at sub-zero temperatures, the design of decontamination interventions at low ambient temperatures requires redesign to prevent hypothermia.

### Surfactant effects

Wester and Maibach (1985) conducted a series of studies looking at the removal of pesticides from the skin of human volunteers (n = 4 to 6) and concluded that in some circumstances (most notably the removal of parathion from the palm), decontamination with hot (temperature not given) soapy water at 15 min post-application increased the penetration of the chemical through skin compared to the 24 hr wash control. Wester and Maibach (1985) suggested that when attempting to remove pesticides with soap and water, the material may in some part spread locally increasing surface area. To investigate this, an additional human volunteer study was conducted comparing a 4 hr post-dose full-body shower to a 4 hr post-dose local wash with soap and water. The results were inconclusive with variations in efficacy for each chemical. Parathion for example was removed more effectively when washed locally with soap and water whereas malathion and the insecticidal formulation Baygon absorption was elevated more when washed locally with soap and water, although no significance was provided for any observation. *In vitro* studies by Moody and Nadeau (1993) demonstrated enhancements in receptor fluid concentrations of DEET following applications to Testskin, rat skin and hairless Guinea pig skin, following a 24 hr post-dose wash with 5 ml of 50% Radiac soap solution (5 ml/min) followed by 5 ml distilled water. Moody and Nadeau (1993) indicated that the comparisons found between the 1985 paper and this study are consistent with the wash-in effect being a result of the “soap liberation of a dermal depot of persisting residues” and not because of mechanical spreading due to showering. In this context, the “dermal depot” relates to the reservoir of chemical that remains within the skin rather than passing through to the receiver solution (Baker et al. 1977; Vickers 1972). Due to the lack of significance between soap washing and showering displayed in the Wester and Maibach study (1985) and the wash-in effect not being identified reproducibly in the 1993 study (no significant effect seen for human skin, and non-reproducible results between individual replicates), it is uncertain whether soap is a major contributor to the wash-in effect. As the investigations did not compare soap water washed to water-only control washes, it is not clear whether soap is exerting an added cumulative effect considering the evidence for simple percutaneous hydration outlined in the previous section.

A series of follow-up studies on the comparisons between *in vitro* and *in vivo* absorption of diazinon (Moody and Nadeau 1994), DDT (Moody, Nadeau, and Chu 1994) and DEET (Moody, Nadeau, and Chu 1995) attributed the inclusion of soap in the decontaminating solution as the primary basis for the wash-in effect. Following the same methodology, the 1994 studies applied diazinon or DDT to a range of *in vitro* skin models including rat, Guinea pig, pig, and human skin. Each study conducted decontamination in the same fashion as the earlier study (5 ml 50% Radiac soap solution at 5 ml/min followed by 5 ml distilled water). Following the 24 hr soap water wash, the amount of penetrated diazinon and DDT increased over baseline values, most pronounced for human skin (recovery over time data was not provided for any other skin models). While the rise was identifiable, the % dose penetrated remained low (human skin baseline for diazinon ≈ 0.3% raising to 0.5% following wash and ≈ 0.01% raising to 0.06% for DDT) suggesting that while the wash-in effect is pronounced, the implication of harm would be low especially considering wet decontamination with soap follows other, effective, soap-free methods of decontamination. Both studies suggest that the elevation in post-wash levels is due to the liberation of the dermal depot due to soap interaction, however no further explanation is given.

A greater magnitude wash-in effect was observed *in vitro* for DEET (Moody, Nadeau, and Chu 1995) when three commercial insecticide formulations of varying DEET concentrations were washed off skin. Following the identical procedure to the 1993 study, Moody, Nadeau, and Chu (1995) noted a 32-fold rise in dermal penetration following a soap water wash compared to pre-decontamination fractions. This temporal increase in DEET penetration was most pronounced when the highest concentration of DEET was used (95% Muskol®) and exceeded the cumulative amount that penetrated in the previous 24 hr. Moody, Nadeau, and Chu (1995) again indicated that this is the depot phenomenon whereby a cutaneous pool of DEET within the SC is released when surfactants affect the skin barrier integrity, through mechanisms such as delipidation or membrane fluidization (Moody and Maibach 2006).

The key methodological flaws with the above studies are the lack of comparisons between soap water washing and clean water washing, and the lack of statistical significance. Wester and Maibach (1985) did not state that there were any significant differences between soap and non-soap water, only their relative performance for the compounds tested. The conclusion that soap was the main contributory factor to the wash-in effect is not clearly evidenced, and subsequently Moody and Nadeau (1997) started to diversify the potential mechanisms for the wash-in effect, even suggesting that the wash-in effect may have been due to simple SC hydration as previously described.

During the study on diethyl malonate, Loke et al. (1999) investigated the effect of both clean water and soap water decontamination solutions *in vitro*. Four different decontaminating solutions (excluding the saline solutions previously discussed but including the deionized water) were perfused (0.5 L/min) over the human skin sample at 0.25, 0.5, and 1 hr post-application of 4 µl diethyl malonate. The solutions were 2% (w/v) sodium dodecyl sulfate (SDS, anionic surfactant), 2% (w/v) hyamine (cationic surfactant), 2% (w/v) RM21 (nonionic surfactant) and deionized water. When compared to the no-decontamination control, all decontaminating solutions displayed an enhancing effect when used to decontaminate the skin at 1 hr post-dosing. The mean penetration rate of the no-decontamination control sample was 99.21 ± 16.46 µg/hr and the mean enhancement in penetration rates were 141.19 µg/hr (SEM 38.6) for SDS, 138.03 µg/hr (SEM 76.06) for hyamine, 135.32 µg/hr (SEM 29.35) for RM21, and 105.55 µg/hr (SEM 30.77) for deionized water, all conducted in triplicate. While there was no marked differences between decontamination interventions and control, there was a trend toward surfactant solutions producing a greater enhancement to penetration over water alone. Data indicate that surfactants may contribute to facilitating the wash-in effect, but it is likely to be one of the multiple contributing mechanisms and further appropriately powered investigations are required to confirm this.

Forsberg et al. (2020) found no marked differences between the cumulative penetration of five physicochemically diverse compounds when decontaminating human skin *in vitro* following rapid decontamination using 10 × 50 µl aliquots of either water or 2% soap solution (DAX soap). Decontamination conducted at 5, 15, 45, and 120 min, was effective for acrylonitrile, 2-butoxyethanol, ethyl lactate, methyl salicylate and tributylamine with no difference in cumulative penetration between water and soap water. No marked change was detected in the cumulative penetration of 2-butoxyethanol for either decontamination solution conducted at 120 min against the no-decontamination control. Forsberg et al. (2020) noted a temporary increase in the concentration of 2-butoxyethanol penetrating skin when soapy water was used, temporarily bringing the penetration rate higher than the no-decontamination control values. Unfortunately, no data was provided for this rise, as results outside of graphs were provided as total cumulative penetration only. It was proposed that the soap is initiating this temporal rise as the same phenomenon was not observed with water decontamination. However, methyl salicylate underwent a temporal increase in penetration rate following both soapy and non-soapy water decontamination, indicating that water may simply be the controlling mechanism for the wash-in effect.

Both Misik et al. (2012) and Thors et al. (2020) also partly attributed instances of the wash-in effect for paraoxon and VX, respectively, to surfactants. Thors et al. (2021b) identified a significant increase in *in vitro* penetration rate of neat VX (1 µl) through human skin (50 min post-exposure onwards) when decontaminating with soapy-water (initiated 5 min post exposure) and compared to a no decontamination control. There was also a significantly larger cumulative penetration between soapy-water wash (0.87 ± 0.18 µg/cm^2^) and the control condition (0.26 ± 0.07 µg/cm^2^) (both n = 4), despite there being a significant decrease in the amount of VX identified in the SC following soapy-water intervention compared to controls. Thors et al. (2021b) postulated that the wash-in effect was primarily due to the soapy-water wash releasing the dermal depot of VX, and removal from the application site prevented reservoir replenishment. Importantly, evidence indicated that this increase in cumulative VX penetration is sufficient to enhance the probability of the onset of cholinergic symptoms, resulting in a shorter timeframe for initiation of medical treatment following exposure. The combination of realistic intervention timings (5 min), and volumes and duration of wet decontamination (10 × 50 µl of 2% soapy water washed and removed in 2 min) suggest that the wash-in effect identified here may well translate under realistic chemical incident conditions.

### pH effects

Of the 16 papers identified in this review, two describe pH as being a possible contributor toward the wash-in effect. Forsberg et al. (2020) speculate that “pH changes” during soap water washing may have been the underlying factor to enhanced penetration but do not provide any further evidence. Moody and Nadeau (1997) however state that the pH of the soap used may influence the penetration of certain chemicals. Through all above studies by Moody and Nadeau (1997), Radiacwash soap is used which when diluted 1:1 in these studies has a reported pH of 4.65. Evidence indicated that there is an expectation for skin permeability to be affected for ionizable pesticides such as the 2,4-D amine utilized in this investigation.

The pH of skin is between 4 and 6, slightly acidic, and it is generally accepted that the acid mantle within the hydrolipid film coating the epidermis is the cause. Endogenous factors such as age and sebum as well as exogenous factors such as cleansing agents may also markedly affect the skin pH. Skin’s purpose is to neutralize alkaline compounds such as harsh surfactants, to allow optimal conditions for skin flora to thrive and to allow the SC to repair itself when damaged. The slightly acidic nature of the skin creates a gradient with the body’s generally neutral internal environment, and this gradient can be easily disrupted by adjusting the pH of the surface of the skin. It is thought that surfactants might alter the pH of the skin surface significantly, initiating a change in barrier function of the SC, and possibly leading to enhanced diffusion of certain chemicals. While this would not be a factor of the wash-in effect alone, it may contribute to a co-enhancement depending on the type of surfactant used.

It is clear that chemical influences on the barrier function of the skin and percutaneous penetration are likely to be contributing to the wash-in effect. The surfactant-enhanced penetration of diethyl malonate (Loke et al. 1999) and the differing absorption kinetics of 2-butoxyethanol with water and soap water is suggested by Forsberg et al. (2020). These investigators also bring into question whether the wash-in effect is chemical-dependent. The wash-in effect is not seen equally across physicochemically diverse compounds as a result of varying thermodynamic activities governing skin penetration and diffusivity across the skin. Data suggest the magnitude of the wash-in effect may be directly linked to physicochemical properties such as lipophilicity of the compound or its pKa which determine the availability of hydrophilic ionized or lipophilic neutral species dependent on the pH of the skin surface.

### Physicochemical effects

While it is well known that certain physicochemical properties influence the rate of penetration (e.g. LogK_ow_ and molecular weight) (Barratt 1995; Ghafourian and Fooladi 2001; Patel, ten Berge, and Cronin 2002; Potts and Guy 1995) no study has specifically examined only the physicochemical characteristics of chemicals and their effect on the wash-in effect. Zhu et al. (2015) investigated penetration in compounds with similar molecular weights and lipophilicities to high toxicity threat agents. When the soap water wash condition was compared to the non-wash condition, a significant decrease in total penetration (1.4–0.5%) was found for hydroquinone (Mw 110.11, LogP 0.59), no significant difference was noted for benzoic acid (Mw 122.12, LogP 1.87) or clonidine (Mw 230.09, LogP 1.59), and a significant rise in receptor fluid concentration (1.2–3.1%) for paraoxon (Mw 275.2, LogP 1.98). While benzoic acid showed no significant difference in total penetration, the rate of penetration temporarily rose significantly when subjected to a soap water wash compared to no-wash control. Hydroquinone is the most hydrophilic compound tested in this study, which may explain why it was so effectively removed by the wash conducted at 30 min post application, however the amount removed with the wash was comparable to all other chemicals tested, and in fact hydroquinone was found at a higher % dose within the epidermis than the other chemicals. When all test chemicals were applied to skin of varying levels of hydration (35%, 174%, and 295% of the dry sample weights) it was found that hydroquinone and clonidine displayed absorption rates disproportionate to the elevation in SC water content, while benzoic acid and paraoxon displayed a correlation in enhanced absorption as the SC water content increased. These findings agree with data demonstrating that benzoic acid and paraoxon display wash-in effects while hydroquinone and clonidine did not; however, while hydrated skin may alter diffusion of the compounds across the skin, surface applied water is responsible for the initial skin penetration. While molecular weight exerts no obvious effect on penetration, increasing LogK_ow_ seemed to be a physicochemical property governing a chemicals ability to be washed in, potentially due to the higher thermodynamic activity of lipophilic compounds in a water carrier.

Contrary to these findings however, a previous human study found that compounds from the same chemical family with varying LogK_ow_ and water solubilities (methyl salicylate – LogK_ow_ 2.5, sol. 0.7 mg/ml; ethyl salicylate – LogK_ow_ 2.95, sol. 0.73 mg/ml; and glycol salicylate – LogK_ow_ 0.52, sol. 12700 mg/ml) display varying degrees of dermal absorption rate when subject to occlusion (and therefore increased skin hydration and rise in thermodynamic activity through saturation of vapor). Wurster and Kramer (1961) showed that compared to non-occluded skin, occluded skin had increased permeation of the three esters by 3.2-, 2-, and 9-fold, respectively, with the most hydrophilic displaying the highest magnitude of penetration. More recently, this study was further investigated *in vitro* by Yousef et al. (2017) who applied 700 µl aliquots of neat salicylates with and without 375 µl of water to represent hydrated and non-hydrated conditions to human skin. The Franz type diffusion cells were capped to avoid compound evaporation. The main findings achieved good correlation with the study by Wurster and Kramer (1961) whereby both methyl and ethyl salicylate increased total penetration over 24 hr two-fold under hydrated conditions and the highly water-soluble glycol salicylate was elevated 10-fold under hydrated conditions compared to dehydrated conditions. The results from these studies on salicylates, and the previous study by Zhu et al. (2015) seem to demonstrate that compounds display enhanced penetration only at particular LogK_ow_ values, possibly due to thermodynamic activity in an aqueous vehicle, however there is no discernible pattern present. It is assumed that compounds with particularly high (> 4) or low (< −1) LogK_ow_ values are expected to penetrate the skin to a lesser extent than compounds within that range (EFSA PRP Panel 2011; EFSA 2017), however as the values selected are arbitrary, and the above investigations show little correlation, LogK_ow_ may not be the most reliable predictor of enhanced dermal penetration (OECD 2019).

While other studies focussed on simple hydration mechanics being the causative mechanism for the wash-in effect, mechanistic evaluations on SC solubility were conducted within the study by Yousef et al. (2017). Hydrated and dehydrated skin samples were immersed in neat solutions of salicylates, and, the uptake of the salicylates into the skin remained unchanged. Results suggested that the enhanced penetration was initiated by an elevation in diffusivity of the salicylates, rather than SC solubility which was an order of magnitude greater for neat penetrants compared to aqueous solutions. Yousef et al. (2017). added that due to glycol salicylates hygroscopicity, once in the SC it may effectively further hydrate the SC by promoting water uptake into the SC, resulting in SC swelling and leading to higher diffusivity. As this was not detected for other salicylates, it is conceivable to discount any other reasons for this marked increase. In this circumstance, the glycol salicylate is proposed to be acting as a hygroscopic humectant, effectively chemically bonding with, and drawing in water, which may enhance SC intercellular fluidity. Other investigators examined the effect of compounds on thermodynamic activity and found that when high concentration solutions or neat compounds are applied to skin, penetration rates significantly fell due to a reduction in water activity and dehydration of skin. Nicotine was shown to elicit a peak flux at a concentration of 48%, and at higher concentrations the epidermal penetration rates decreased (Kuswahyuning and Roberts 2014). This was also reported for 2-butoxyethanol (Bunge, Persichetti, and Payan 2012), whereby a reduction in water activity from 0.9 at 80% concentration, to zero at pure 2-butoxyethanol correlated with a decrease in epidermal flux. In the salicylate study by Zhu et al. (2015) found that 700 µl glycol salicylate displayed a 10-fold enhancement when co-applied with 375 µl water. The enhancement in penetration under hydrated conditions may be due to both thermodynamic activity within the aqueous vehicle, and an elevation in diffusivity as a result of hygroscopic action.

A further chemical-specific finding in this study was the lower than expected penetration enhancement for methyl salicylate *in vitro* compared to Wurster and Kramer (1961). In humans, occlusion resulted in a 3.2-fold increase in penetration while *in vitro* the enhancement was only twofold. Zhu et al. (2015) proposed this discrepancy is because of the rubefacient properties of methyl salicylate, that would only be apparent on living skin. Methyl salicylate is used as a topical analgesic and increases local blood flow and temperature of the skin. Both mechanisms are known to increase diffusivity of the SC which would explain the greater penetration *in vivo*.

The penetration rate of 10 µl VX through porcine skin *in vitro* (Dalton, Graham, and Jenner 2018) was transiently significantly higher between 1 and 3 hr post-application, when applied in a finite dose to wet skin compared to dry skin. There was no significant difference in penetration rates between 12 and 24 hr. Under *infinite* dosing test conditions, the steady state penetration rate for 50% VX (aqueous solution, 500 µl total application) was 366 ± 149 µg/cm^2^ hr, significantly higher than observed for 500 µl neat VX (169 ± 89 µg/cm^2^ hr). It was tentatively speculated that the rise in steady state penetration rate was possibly due to changes to both diffusivity of the SC and diffusional path length. A higher diffusion coefficient and a lower diffusional path length would both result in an increase to the steady state according to Fick’s Law. This assumption also infers that undiluted solutions of agents may exert the reverse effect by potentially dehydrating the skin, reducing the permeability coefficient and therefore epidermal flux, as previously shown with nicotine and 2-butoxyethanol. Thors et al. (2020) postulated that the addition of water *in vitro* to neat VX may have formed a concentration gradient across the skin, enhancing the permeation of VX. In their study, rather than dilute VX in water, the cell was decontaminated with 10 × 50 µl of 2% soapy water. Decontamination occurred at 5 min post-exposure and lasted 1 min. The cumulative amount of VX penetrated over 5 hr after wet decontamination was significantly higher (122.4 ± 22.3 µg/cm^2^) than through non-decontaminated control condition skin at 56.0 ± 6.7 µg/cm^2^ (Mean ± SEM; both n = 6). The penetration rate through skin following wet decontamination was also significantly higher than control conditions, peaking at around 1.5 hr post-decontamination.

### In vitro artifacts

*In vitro* investigations provide certain benefits over *in vivo* studies when examining specific mechanisms such as the wash-in effect. *In vivo* investigations may (1) be very costly, (2) include rigorous animal husbandry requirements, and (3) provide more complex results to interpret. *In vitro* experiments on the other hand focus on one particular mechanism at a time without confounding variables and may be relatively simple and cost-effective to conduct. *In vitro* studies are not without its drawbacks however, including the sometimes-unrealistic experimental conditions that distance the results from what would be expected in reality. This might lead to possible over- or under estimations of values.

While both Thors et al. (2020) and Dalton, Graham, and Jenner (2018) identify the same effect, the methodology varies slightly in the receptor fluid used. Dalton, Graham, and Jenner (2018) employed ethanol:water (1:1 v/v) while Thors et al. (2020) used ethanol:water (1:3 v/v). The receptor fluid utilized previously by Thors et al. (2016) identifying the penetration of various lipophilic and hydrophilic organophosphorous compounds (OPCs) found that 1:1 concentrations of ethanol in the receptor solution significantly increased penetration of 20% diluted OPCs over receptor fluids with 1:3 or 1:9 ethanol: water. Data suggest that the magnitude of the wash-in effect reported by Thors et al. (2020) and Dalton, Graham, and Jenner (2018) may be overestimating the magnitude of the wash-in effect. There is debate as to whether 1:1 ethanol:water receptor fluid compositions are suitably representative to *in vivo* conditions (Ramsey et al. 1994; Saunders and Pugh 2002), and whether it may change the hydration of the SC and the diffusion of compounds into the SC through the ethanol concentration gradient formed, overestimating the wash-in effect *in vitro* (Jones, Greenway, and Orr 1989). Yousef et al. (2017) commented on the confounding influence of receptor fluid composition on the quantity and mechanism of skin permeation. Using a 6% Volpo N20 in phosphate buffered saline receptor fluid Yousef et al. (2017) showed that it did not confound the dermal penetration of the compounds but provided appropriate sink conditions. Finding the correct receptor fluid for a particular study can be challenging, especially when comparing compounds with diverse physicochemical properties or where static diffusion cells are employed. Aqueous receptors are commonly used for hydrophilic and moderately lipophilic compounds while more lipophilic compounds require solubilizing agents and additives such as organic solvents or proteins. Regardless of the compounds of interest, the further the receptor fluid is from *in vivo* conditions greater uncertainty for physiological relevance. The *in vitro* studies identified here used either ethanol:water mixtures or buffered salt concentrations with and without serum. For this reason, it cannot be ruled out that the wash-in effects identified in these papers may be artefactual, at least in part attributed to the receptor fluid composition and the considerable methodological differences between studies makes comparison difficult.

Another potential *in vitro* artifact is the dosing solutions employed. Studies mostly apply the compound of interest neat, in an aqueous solution, or in a formulation such as sunscreens or moisturizing lotions, while those by Moody and Nadeau (1993, 1994, 1997), Moody et al (1994, 1995) and Wester, Noonan, and Maibach (1977) used acetone as the diluent. It is important that the appropriate carrier is utilized as it will have direct effects on skin permeation. For relatively insoluble compounds such as diazinon, DDT and 2,4-D used by Moody *et al*.(1994, 1995) aqueous carriers would be ineffective which may suggest why acetone was used. Acetone is known to interact with the structure of the skin *in vivo* and adjust the permeability of the barrier (Benfeldt and Serup 1999), in particular the ingress of hydrophilic compounds (Tsai et al. 2001). Tape stripping and soap naturally decreased the barrier integrity and produced a significant rise in penetration of salicylic acid. Acetone treatment (gentle wiping with cotton ball soaked in acetone 20 min prior to compound application) might selectively remove lipids from the intercellular domain and increase trans epidermal water loss (TEWL), however an overall increase in barrier function was detected by a reduction in salicylic acid permeation over the control group. It was postulated that had the acetone treatment been more intense and prolonged it would have resulted in an increase in skin penetration of salicylic acid, although provided no clear evidence was provided. It is evident that use of acetone may have an impact on the wash-in effect if used as the carrier solution. It seems sensible that compounds of interest need to be applied in a way that is appropriate to the research question being addressed. For consumer products, this may include addition as a lotion or with soap water if used in cleaning products for agrochemicals this may be in the appropriate formulation intended for utilization in the field.

The various receptor fluids employed and use of solvent as a carrier support claims that the wash-in effect is influenced by *in vitro* artifacts due to methodological design (Moody and Maibach 2006), however the specific contribution toward the wash-in effect is difficult to determine without further primary research.

An additional *in vitro* artifact comes from a lack of contact between the underside of the skin and the receptor fluid (Moody and Nadeau 1997). For an *in vitro* study to be conducted effectively, a constant contact between the receptor fluid and the skin is required. Any trapped air bubbles might result in an accumulation of penetrant on the underside of the skin, which when reunited with the receptor fluid might initiate an apparent “burst” of penetration. Moody and Nadeau (1997) discounted this as the mechanism behind the wash-in effect observed during their study, citing unpublished data that no bubble was observed. Further, more modern *in vitro* chambers have very small receptor fluid compartments, reducing the likelihood of bubble formation, and the turbulence produced by stirring bars or by the receptor fluid flow itself or skin contact during active decontamination stages may also create or dislodge bubbles. Dislodging bubbles during an experiment would reunite the skin and receptor fluid and possibly initiate a sudden, temporal increase in skin permeation. No additional studies identified in this review discuss the possibility of bubbles.

Previously Misik et al. (2012) spread out paraoxon on showered skin while it stayed in a droplet on untreated control skin. This had the unintended consequence of the dose spreading out not only over the skin and thereby increasing effective penetration area but also between the edge of the skin samples and the modified Franz diffusion cell walls. This may have created a reservoir of dosing agent that bypassed the skin during decontamination. While this phenomenon is only highlighted by Misik et al. (2012) it cannot be ruled out for other investigations using Franz diffusion cells that identified the wash-in effect after either pre-wetting, or using enough water to carry chemicals to the edge of the skin sample and chamber.

Experimental design as well as apparatus might also influence the wash-in effect. Matar et al. (2016) assessed efficacy of military, commercial and novel products to decontaminate methyl salicylate *in vitro*. One objective was to identify a suitable replacement for water that first responders can employ in a mass casualty decontamination event that does not elicit a wash-in effect. Results demonstrated that compared to the no intervention control condition, interventions containing water including diphoterine and baby wipes significantly enhanced both dermal absorption and maximum penetration rate, whereas medical moist-free wipes and Florafree solution both significantly increased maximum penetration rate only. Matar et al. (2016) suggested that the water in diphoterine and the FloraFree solution, and the surfactants in the baby wipes may have elicited this wash-in effect. Five min following application of the methyl salicylate, the decontamination interventions were largely conducted in line with the UK IOR (Home Office 2015) but in this study, instead of conducting decontamination at 15 min, all interventions were left *in situ* for 24 hr. Occlusion of skin might enhance penetration of compounds, and non-water-soluble, volatile methyl salicylate would have been trapped on the skin increasing the probability of penetration. Wet decontamination including with diphoterine is conducted as a rinse method, rather than immersion. It is perhaps surprising therefore that greater significant increases in penetration were not reported. This issue may also be present in other experiments within this review, specifically those that did not factor in either evaporative loss of small volumes of penetrants or air flow surrounding the *in vitro* cell that might affect skin hydration, both of which reduce the likelihood of producing data that would agree with *in vivo* studies.

The final potential *in vitro* artifact concerns the differentiation of bioavailable and non-bioavailable fractions of a compound within the skin. *In vivo* studies rely on biomarkers (the chemical applied or its metabolites) in urine, feces, or blood to determine the dermal penetration of chemicals. While *in vitro* studies replicate this through the time coursed sampling of receptor fluid to determine the rate of penetration, investigations that determine cumulative penetration often analyze both the penetrated fraction and the fraction remaining in the skin. The fraction within the skin is deemed bioavailable as it is likely it will continue penetrating the skin, and while the inclusion of residues within the dermis generally results in reliable *in vivo – in vitro* agreement (Ross, Reifenrath, and Driver 2011), the addition of the SC residues may result in an overestimation of *in vitro* results when compared to comparable *in vivo* results (Reifenrath 2012). Moody, Nadeau, and Chu (1995) described this artificial enhancement when their reported *in vitro* data significantly overestimated the *in vivo* data. Further research needs to identify whether studies that report higher cumulative penetration of compounds through skin *in vitro* when compared to *in vivo* studies are seeing higher levels purely due to the inclusion of the bioavailable fraction within the skin itself.

### Relevance of the wash-in effect to mass decontamination

From the available literature, it is clear that there is a limited body of work examining the wash-in effect specifically, and none that investigated all the proposed mechanisms, including whether it is an *in vitro* artifact. It is possible that the wash-in effect is not due to a single mechanism, but rather a combination of mechanisms with the contribution of each dependent upon the physicochemical characteristics of each chemical and the experimental conditions.

The possibility of a wash-in effect has led to a general wariness and caution with regards to operational policy around wet mass decontamination protocols. As part of the Optimization through Research of Chemical Incident Decontamination Systems (ORCHIDS) project, the UK model response to a chemical incident as outlined by the Home Office was changed to include first responder priorities, interim methods of decontamination and adjustments to SOR. Among these adjustments was the recommendation that the showering duration within a mass decontamination unit (MDU) be reduced from 3 min to 90 sec. This was in part to increase the throughput of the MDU, increasing the number of casualties able to be decontamination per hour, but also as a reaction to the possibility that extended shower duration and water exposure *could* lead to a wash-in effect (Amlot, Riddle, and Chilcott 2011; Collins et al. 2021a). While this fundamental change to operational guidance in the UK was made on a precautionary basis, further evidence demonstrated that this reduction in showering time did not have a significant deleterious impact on the efficacy of decontamination based upon chemical remaining on skin. Further work is required to determine whether this is also true for systemic exposure, though initial work conducted under the Protocols for Hair and the Optimization of Existing and Novel decontamination Interventions through eXperimentation (PHOENIX) project was unable to demonstrate a significant reduction in urinary levels of either methyl salicylate or benzyl salicylate in a series of human volunteer decontamination studies following skin and hair application despite significant reductions to levels remaining on hair and skin (Collins et al. 2020, 2021b; Southworth et al. 2020).

Three of the studies identified in this review (Loke et al. 1999; Misik et al. 2012; Zhu et al. 2015) conducted wet decontamination within 1 hr of chemical exposure, lasting between 1 and 9 min. In mass casualty events, interim decontamination typically lasts approximately 60 sec while mass decontamination is normally conducted for 3 min, with only 90 sec of contact with warm soapy water. The corroboration between the *in vitro* study designs and the operational timings involved in mass decontamination give weight to the plausibility of a wash-in effect in humans. It is important to consider however, that in the UK, IOR recommends that if the chemical is non-caustic, dry decontamination is always conducted first. Typically, dry decontamination involves blotting the skin with dry absorbent materials, significantly reducing the levels of agent on the skin by up to approximately 85% (Southworth et al. 2020) prior to any exposure to water. This is then followed by wet decontamination methods including interim decontamination with ladder-pipe systems, then SOR. As such, the bulk of contamination should have already been removed through disrobing and dry decontamination, before the skin is subjected to washing. Few of the identified studies investigate the potential wash-in effect in the context of mass casualty operational response where dry decontamination, precedes wet decontamination with cold water (interim decontamination) and only warm water and detergent (MDU) as a final step. It is clear that the relevance of the wash-in effect during mass casualty decontamination following a chemical incident cannot simply be influenced by *in vitro* data alone.

The contribution of the wash-in effect to systemic levels of chemicals in the context of casualty mass decontamination is unknown. Specific study design would be required to better understand the wash-in effect in context, including an initial reduction of surface contamination using dry decontamination which may possibly increase skin contamination surface area aiding dermal absorption, decontamination methods using a proportionally appropriate volume of water to mimic interventions such as ladder pipe systems or MDUs, and timely collection of a biologically appropriate receptor fluid. Josse et al. (2011) described an *in vitro* study into the wash-in effect under more appropriate conditions, focussing on dry decontamination and pre-shower treatments for removal of VX, and the cumulative effect of the conducting wet decontamination. Dry decontamination methods were implemented 30 min post-dosing, and 1 min showering followed 60 min post dose. Based on the amount of VX that had penetrated the skin 6 hr post dose, Josse et al. (2011) found no evidence of a wash-in effect.

The possible transient rise of dermal absorption in humans is an interesting concept, however what is required is an understanding of the health consequences of this increase. Modeling could be conducted to determine the potential health consequences of transient enhanced systemic exposure due to the wash-in effect. If enhanced levels of penetrated compound following national guidelines for decontamination are sufficiently small, then the real-world consequences of the wash-in effect for mass casualty decontamination may be negligible except for the most highly toxic chemicals. What is important then is that casualties are decontaminated sufficiently to enable them to receive medical interventions without endangering first-responders or clinicians.

A particular concern is the decontamination of powders, which do not normally penetrate skin unless solubilized. While IOR guidance in the UK recommends dry decontamination for powders (unless corrosive, radiological or biological) (Home Office 2015), US guidance recommends wet decontamination is used to decontaminate powders (Chilcott et al. 2019). This direct contradiction between UK and US guidance shows that further research is required to fully understand the wash-in effect and how it may impact operational guidance.

A lack of harmonization between *in vitro* studies and the varied methodologies employed, prevents convincing comparisons between these investigations and the potential mechanisms under investigation being drawn. To better understand exactly what causes the wash-in effect, and how to mitigate its potential occurrence during chemical mass casualty decontamination, future work needs to focus on the different mechanisms under realistic conditions. A study needs to consider comparisons between wet decontamination both with and without soap, with and without active washing, and the effect of different types of soaps with different pH. These should be compared to appropriate baseline controls and should (1) employ realistic receptor and donor fluids, (2) appropriate timings and methods for decontamination interventions, (3) appropriate chemical doses and timely collection methods. The investigations would need to factor in physicochemical diversity of compounds including vapor pressures, LogK_ow_, pKa, water solubilities, and molecular weights.

The *in vitro* and *in vivo* studies reviewed above showed there are merits to data collection on the wash-in effect using either method, be it the cost-effective nature and specificity of mechanism afforded by *in vitro* studies, or the biological interactions present in *in vivo* studies. Both, however, have their drawbacks, including the possibility of an *in vitro* artifact influencing the wash-in effect, and no *in vivo* test being fully representative of human pharmacokinetics or physiology (Bronaugh and Maibach 2005; Burli et al. 2021). To better understand the real-world relevance of the wash-in effect for mass decontamination, the goal needs to be to identify the wash-in effect using simulants in human volunteers. These studies need to include appropriate sample collection methods to detect temporal effects, along with a suitable range of physicochemically diverse chemicals applied to various sites on the body to normalize for variations in skin permeability and hydration. With better understanding of the wash-in effect under these conditions, mass decontamination interventions may be fine-tuned to work more effectively in response to a chemical incident.

## Conclusions

Despite *in vitro* artifacts contributing to the wash-in effect, the literature identified has provided compelling evidence for a wash-in effect. Most studies were conducted *in vitro*, and six potential contributory mechanisms were described. The wash-in effect is likely a combination of these effects that each contributes to enhanced penetration in a chemical-specific manner. The observations are dependent upon study conditions, experimental protocol and are influenced by the aims and objectives of each study. Studies focussed on wet decontamination for example, focus on hydration of the SC leading to a change in structure that facilitates chemical penetration, the effect of a surface water carrier on the initial penetration into the SC, or that detergent enhances dermal penetration through surfactant effects. *In vitro* studies are more likely to additionally refer to mechanical effects contributing to the wash-in effect such as desquamation of skin through excessive washing or force, compromising the barrier properties of the SC, or spreading of the chemical over a larger surface area of skin, leading to increased penetration. It is essential to clearly address the uncertainties around the wash-in effect and mass casualty decontamination, determine the real-world consequences of wet decontamination methods to the systemic bioavailability of physicochemically diverse hazardous materials or appropriate simulants, and inevitably conclude whether mass casualty intervention strategies need to be modified to achieve the best outcomes for casualties following a chemical incident.

## Data Availability

Data sharing is not applicable to this article as no new data were created or analyzed in this study.

## Appendix

**Appendix A** – Full literature searches as of 24/07/2020. Additional references identified through automatic monthly update up to 02/12/2021 not included.

***Search 1* Medline (181 results) (1946 – July 2020: in -process, in-data review and other non-indexed citations)**.

**Table:**

1	Skin Absorption/	11678
2	skin penetra*.tw.	2131
3	dermal penetra*.tw.	286
4	percutaneous absor*.tw.	2079
5	skin absor*.tw.	708
6	dermal absor*.tw.	971
7	skin diffus*.tw.	119
8	dermal diffus*.tw.	23
9	1 or 2 or 3 or 4 or 5 or 6 or 7 or 8	14169
10	enhanc*.tw.	1471408
11	9 and 10	3512
12	(skin or dermal).tw.	554169
13	(hydrat* or wash*).tw.	183811
14	12 and 13	7204
15	11 and 14	181

## Appendix

**Scopus (482 results) (no restrictions)**

((ALL (enhance*) W/2 ALL (skin OR dermal OR percutan*) W/2 ALL (penetra* OR absor* OR diffus*)) AND (ALL (skin OR dermal) W/2 ALL (hydrat* OR wash*)) AND (chemical))

**EMBASE (5 results) and PubMed (58 results) through HDAS (no restrictions)**

(enhance*) ADJ2 (skin OR dermal OR percutan*) ADJ2 (penetra* OR absor* OR diffus*) AND (skin OR dermal) ADJ2 (hydrat* OR wash*) AND (chemical).

**Total references as of 24/07/2020 for main search = 726**

***Search 2***

**Medline (9 results) (1946- July 2020: in -process, in-data review and other non-indexed citations)**.

**Table:**

1	“Wash-in effect”	9
2	“Wash in effect”	9
3	1 or 2	9

**Scopus (79 results) (no restrictions)**

(“Wash-in effect” OR “Wash in effect”)

**EMBASE (10 results) and PubMed (9 results) through HDAS (no restrictions)**

(“Wash-in effect” OR “Wash in effect”)

**Total references as of 27/07/2020 for secondary search = 107**
